# HnRNPC triggers the degradation of MITA to suppress the interferon-mediated antiviral response

**DOI:** 10.1186/s13567-025-01463-6

**Published:** 2025-02-24

**Authors:** Yanwei Zhang, Zhao Jia, Gaoliang Yuan, Kangyong Chen, Jing Cen, Junya Wang, Hao Feng, Mikolaj Adamek, Jun Zou

**Affiliations:** 1 Key Laboratory of Exploration and Utilization of Aquatic Genetic Resources, Ministry of Education, Shanghai Ocean University, Shanghai, 201306 China; 2 Laboratory for Marine Biology and Biotechnology, Qingdao Marine Science and Technology Center, Qingdao, 266200 China; 3 International Research Center for Marine Biosciences, Ministry of Science and Technology, Shanghai Ocean University, Shanghai, 201306 China; 4 School of Fisheries, Xinyang Agriculture and Forestry University, Xinyang, 464000 China; 5 State Key Laboratory of Developmental Biology of Freshwater Fish, College of Life Science, Hunan Normal University, Changsha, 410081 China; 6 Fish Disease Research Unit, Institute for Parasitology, University of Veterinary Medicine Hannover, Hannover, Germany

**Keywords:** HnRNPC, interferon, antiviral response, MITA, ubiquitination

## Abstract

Heterogeneous nuclear ribonucleoproteins (hnRNPs) are a group of 34–120 kDa nuclear proteins that have recently been reported to participate in virus replication. The hnRNP family contains approximately 20 members, including hnRNP A1, hnRNP A2, hnRNP A2B1, hnRNPC, hnRNPD and hnRNPK. HnRNPC plays important roles in RNA biology, including expression, stability, mRNA splicing, nonspecific sequence export and 3’-end processing; however, the mechanisms underlying hnRNPC regulatory roles are not fully understood. Here, we found that zebrafish hnRNPC promoted spring viraemia of carp virus (SVCV) replication by increasing the stability of SVCV phosphoprotein while inhibiting the K48-linked ubiquitination of virus phosphoprotein, thereby suppressing the type I interferon (IFN) response. Mechanistically, hnRNPC could interact with the mediator of IFN regulatory factor 3 activation (MITA) to activate K48-linked ubiquitination for MITA degradation through the C-terminal domain of hnRNPC. We also showed that human hnRNPC could interact with MITA and that the overexpression of human hnRNPC decreased MITA protein in HEK293 cells, suggesting that the negative regulatory effects of hnRNPC on the type I IFN response are evolutionarily conserved. Collectively, our data indicate that hnRNPC promotes virus replication by suppressing IFN production activated by MITA and increasing the availability of viral proteins. Our work reveals an evolutionarily conserved mechanism that controls the IFN-mediated antiviral response by a member of the hnRNP family in vertebrates.

**Supplementary Information:**

The online version contains supplementary material available at 10.1186/s13567-025-01463-6.

## Introduction

The interferon (IFN) system is a major component of innate immunity and plays an important role in host resistance to viral pathogens [1]. It is activated by a cascade of pattern recognition receptors (PRRs), including Toll-like, NOD-like, and RIG-I-like receptors (RLRs), following the recognition of pathogen-associated molecular patterns (PAMP) [2]. RLRs include RIG-I, melanoma differentiation-associated gene 5 and the laboratory of genetics and physiology 2 and are major cytoplasmic PRRs that sense viral RNA PAMPs to trigger innate immune response to inhibit viral replication [3]. Upon sensing viral RNA, RIG-I and MDA5 recruit the signal adaptors mitochondrial antiviral signalling protein (MAVS, also called VISA, IPS-1, and Cardif) to activate TANK-binding kinase 1 (TBK1) and inducible nuclear factor kappa B (IκB) kinase [4–7]. Subsequently, TBK1 and IKKε phosphorylate IFN regulatory factor 3/7 (IRF3/7) and IκB to induce the expression of type I IFNs and IFN-stimulated genes (ISGs) [8].

The production of type I IFNs is tightly controlled by many factors, including TBK1, mediator of IRF3 activation (MITA), IRF3, and IRF7. The MITA-TBK1-IRF3/IRF7 axis is indispensable for the transcription of IFN genes and is known to be modulated at the posttranslational level by multiple factors from both host and virus [9, 10]. For example, MITA is often targeted by host factors [11–13], such as RING-finger protein 90 which enhances the K48-linked ubiquitination and proteasome degradation of MITA to negatively regulate the production of IFNs [14]. Moreover, ubiquitin-specific protease (USP) 49 has been shown to interact with MITA to block MITA aggregation, thereby inhibiting TBK1-mediated antiviral responses [15], and deubiquitination of USP49 promotes virus replication [16]. In addition to MITA, TBK1-IKK-ε-IRF3 interactions can be impaired by host factors such as the cytoskeletal protein vimentin and IFN-induced proteins, resulting in the inhibition of the phosphorylation and nuclear translocation of IRF3 and reduced IFN production [17, 18]. These findings demonstrate that the network controlling IFN production is complex and that more regulators have yet to be discovered.

Heterogeneous nuclear ribonucleoproteins (hnRNPs) are the most abundant nuclear proteins in higher eukaryotes and play important roles in RNA transcription, pre-mRNA splicing, and translation [19]. The hnRNP family comprises approximately 20, 34–120 kDa proteins, some of which are involved in viral infection [20–22]. It has been shown that hnRNPA1 interacts with the nucleocapsid proteins of many viruses, such as SARS coronavirus (SARS-CoV) [23], porcine epidemic diarrhea virus (PEDV) [24], and mouse hepatitis virus (MHV) [25], to promote viral replication. Additional hnRNPs that favour viral replication include hnRNPA2 [26], hnRNPA2/B1 [27], hnRNPD [28], and hnRNPK [29]. HnRNPC is an RNA-binding protein that is essential for RNA transcription and splicing [30, 31]. Emerging evidence indicates that hnRNPC participates in the regulation of infection of several viruses, including influenza [32], poliovirus [22], dengue virus [33], Ebola virus [34], Middle East respiratory syndrome coronavirus (MERS-CoV) and SARS-CoV-2 [35]. However, the underlying mechanisms by which hnRNPC modulates virus infections have not been fully elucidated.

In this study, we investigated the interactions between hnRNPC and key host transcription factors in the RLR signalling pathway to elucidate the roles of hnRNPC in regulating viral replication in zebrafish. Moreover, the effects of hnRNPC on the stability of viral proteins were evaluated. Our study reveals a novel role of hnRNPC in regulating viral replication and provides mechanistic insights into the interactions between host and virus in lower vertebrates.

## Materials and methods

### Cells and virus

HEK293 cells (human embryonic kidney cell line 293, ATCC CRL-1573), EPC cells (*Epithelioma papulosum cyprinid* cell line, EPC, ATCC CRL-2872) [36], ZF4 cells (zebrafish embryonic fibroblast line, ATCC CRL-2050) and DrFIN cells (from the caudal fin of zebrafish in our laboratory, previously named ZFIN cells) [37] were cultured in a 5% CO_2_ incubator and Dulbecco’s modified Eagle’s medium (DMEM; Gibco, USA) supplemented with 10% foetal bovine serum (FBS; Gibco) and 1% penicillin‒streptomycin (P/S) at 37 °C (HEK293 cells) and 28 °C (EPC, ZF4 and DrFIN cells). Spring viraemia of carp virus (SVCV) was propagated in the EPC cells [38].

### Plasmids

Using cDNA samples from DrFIN cells as templates, the coding region of the zebrafish (*Danio rerio*) *hnRNPC* gene (AAQ97793.1) was amplified by polymerase chain reaction (PCR) and sequenced. The predicted hnRNPC protein contains an RRM1 domain and a C-terminal domain. Truncated mutants, including hnRNPC_RRM_ (containing the RRM1 domain) and hnRNPC_C_ (containing the C-terminal domain), were cloned and inserted into p3 × Flag-cmv-14, while GFP-MITA_N_ and GFP-MITA_C_ were cloned and inserted into pEGFP-N1. Plasmids, including Flag-MAVS, Flag-MITA, GFP-MITA, Flag-TBK1, Flag-IRF3, Flag-IRF7, Myc-SVCV-P, Myc-SVCV-P_N_, Myc-SVCV-P_CD_, Myc-SVCV-P_C_, and pGL3-ifnφ1pro, were previously constructed in our laboratory [38, 39]. ISRE-pro, IFNβ-pro and pRL-TK (Promega, USA) were used. HA-ub plasmid and mutant (HA-K48O and HA-K63O) plasmids (HedgehogBio, China) were used. The gene primers used for cloning are listed in Table 1.

**Table 1:** **Information on the primers used in the study**.

Application	Primer	Sequence (5′-3′)
Plasmid construction	Flag-hnRNPC-FW	CCCAAGCTTATGGCCAGTAATGTCACCAAC
	Flag-hnRNPC-BW	CCCAAGCTTATGGCCAGTAATGTCACCAAC
	Flag-hnRNPC_RRM_-FW	CCGGAATTCATGGCCAGTAATGTCACCAAC
	Flag-hnRNPC_RRM_-BW	CGGGGTACCGAATTAATATCGAGTACCTGTC
	Flag-hnRNPC_C_-FW	CCGGAATTCATGCTGGCTGGCGAGCCCAA
	Flag-hnRNPC_C_-BW	CGGGGTACCGAAGCATGATCTCCGTTGGCAC
qRT‒PCR siRNA	Flag-MITA_N_-FW Flag-MITA_N_-BW Flag-MITA_C_-FW Flag-MITA_C_-BW GFP-SVCV P-FW GFP-SVCV P-BW GFP-MITA_C_-K27R-FW GFP-MITA_C_-K27R-BW GFP-MITA_C_-K45R-FW GFP-MITA_C_-K45R-BW GFP-MITA_C_-K53R-FW GFP-MITA_C_-K53R-BW GFP-MITA_C_-K62R-FW GFP-MITA_C_-K62R-BW GFP-MITA_C_-K100R-FW GFP-MITA_C_-K100R-BW GFP-MITA_C_-K149R-FW GFP-MITA_C_-K149R-BW GFP-MITA_C_-K220R-FW GFP-MITA_C_-K220R-BW ZF-HnRNPC-FW ZF-HnRNPC-BW SVCV-N-FW SVCV-N-BW SVCV-G-FW SVCV-G-BW sihnRNPC-1 sense (5′-3′) sihnRNPC-1antisense (5′-3′) sihnRNPC-2 sense (5′-3′) sihnRNPC-2antisense (5′-3′) sihnRNPC-3 sense (5′-3′) sihnRNPC-3antisense (5′-3′)	CGGAAGCTTATGGAGCAGAAGCTGATCAG CCCCGGTACGACGTTCACCTCTAAAGCTG CGGAAGCTTATGAGAGAATACTCTAGAAGGGA CGCGGTACCGTTTTGTTTCATTGCGCTAG CGGAAGCTTATGGAGCAGAAGCTGATCAG CGGGGTACCAGTCTGTACTTCTGATACA AATGCCAGAGTCCCAAGCCGACCTGGAGAGGAGGAC GCGCTTGGGACTCTGGCATTGAGGGGCAGAAGGAT GAAAACCTTCCGGATCTGCGGCTGGACAGGGCAGG CGCAGATCCGGAAGGTTTTCATGGAAGACCAC ACAGGGCAGGAGTGCGGCGACGCAGCTACACTAA CGCCGCACTCCTGCCCTGTCCAGCTTCAGATC TACACTAACAGCGTCTACCGGATCACCCACAACAA CGGTAGACGCTGTTAGTGTAGCTGCGTTTCCG GTTTGGCGAGAGAGAACGGCGGCAGCAGGTCCTG CGCCGTTCTCTCTCGCCAAACCCTGCACTGCTCTCCT GAGCTCTTCCAGAACCTGCGGCAGCAGGATGG CGCAGGTTCTGGAAGAGCTCTCTGGAGAGGTAATG AACCCATCTAGCGCAATGCGACAAAACGTACCGC GCATTGCGCTAGATGGGTTAAAATAATCGGTGGTCT AGTACGCCAACGAGAGGAAC ATGGTCTTTGACCGGTGAGG TCTGCCAAATCACCATACTCA CTGTCTTGCGTTCAGTGCTC ATCATTCAAAGGATTGCATCAG CATATGGCTCTAAATGAACAGAA GAGGCCAUCUUUAGUAAGUTT ACUUACUAAAGAUGGCCUCTT GGAACUGACUCAGAUUAAATT UUUAAUCUGAGUCAGUUCCTT GACAGGUACUCGAUAUUAATT UUAAUAUCGAGUACCUGUCTT

### Reagents and antibodies

Antibodies for immunoblotting, including α-Flag (0912–1), α-Myc (R1208-1), α-HA (0906–1) and α-GFP (ET1607-31), were purchased from HUABIO (China), and α-β-actin (AC038), were purchased from ABclone (China). Goat α-mouse IgG secondary antibody (925–32,210) and goat α-rabbit IgG secondary antibody (926–32,211) were purchased from LI-COR (USA). MG132 (M126521), 3-methyladenine (3-MA) (M129496) and chloroquine CQ (C193834) were purchased from Aladdin (China). jetOPTIMUS transfection reagent (101,000,006) (Polyplus, China). RIPA buffer (P0013C) (Beyotime Biotechnology, China) was used. α-Myc (M20012) or α-GFP (M20015) affinity gels were purchased from Abmart (USA). SiRNAs targeting hnRNPC (si-hnRNPC-1, si-hnRNPC-2, and si-hnRNPC-3) and control siRNA (siNC) (GenePharma, China) were synthesized, and sequences are described in Table 1.

### Viral infection

SVCV was propagated in the EPC cells until a CPE was observed. The culture medium containing the viruses was collected and stored at -80 °C. DrFIN and ZF4 cells were seeded in 12-well plates for 12 h and infected with SVCV (MOI = 1). The cells were collected at different time points for analysis of *hnRNPC* and *mx* gene expression by quantitative real-time PCR (qRT-PCR). For transfection, EPC cells were seeded in 12-well plates and transfected with 1 μg of Flag-hnRNPC or empty plasmid. Twenty-four hours later, the cells were incubated with SVCV [39] at 25 °C for 2 h, and the inoculum was removed. The cells were washed twice with PBS, followed by the addition of fresh DMEM containing 5% FBS. After an additional 48 h, the media were collected for virus titration using the TCID_50_ assay. Briefly, the media were diluted from 10^–1^ to 10^–8^, respectively, and added to the EPC cells in 96-well plates, and after 3 days, the cells were fixed with 4% paraformaldehyde and stained with crystal violet to visualize the cytopathic effect (CPE) [38].

### qRT-PCR analysis

RNA was reverse transcribed into cDNA by mixing 1 µg of RNA with 1 µL of gDNA digester (Yeasen, China), 2 µL of 5 × gDNA digester buffer, and RNase-free H_2_O in a reaction volume of 10 µL. The mixture was incubated at 42 °C for 2 min. Ten microliters of 2 × Hifair^®^ II SuperMix Plus was added, and the mixture was then incubated at 25 °C for 5 min, at 42 °C for 30 min, and at 85 °C for 5 min (Yeasen, China). qRT-PCR was performed with Hieff UNICON power qPCR SYBR Mix (Yeasen) and run on a LightCycle 480 Real-Time System (Roche). β-actin was used for normalization of expression [39]. Fold changes were calculated by comparing the average expression levels of the experimental group to that of the corresponding groups (defined as 1). The primers used are listed in Table 1.

### Western blotting

The cells were collected and lysed with RIPA lysis buffer (Beyotime, China). The samples were separated on a 12% SDS-PAGE gel and transferred onto a polyvinylidene difluoride membrane. (PVDF) membranes (Biosharp, China). The membranes were subsequently blocked with Tris-buffered saline (TBS) containing 5% skimmed milk at room temperature for 2 h, followed by three washes with TBS containing Tween-20 (TBST) and incubation with the designated primary antibodies for 2 h. Then, the membranes were washed three times with TBST buffer and incubated with the secondary antibodies for 1 h. The membranes were examined under an Odyssey CLx Imaging System (LI‒COR) and photographed.

### Coimmunoprecipitation assay

HEK293 cells were seeded in 25 cm^2^ cell culture flasks overnight and transfected with 10 μg of plasmids as indicated in the figures. Twenty-four hours post-transfection, the culture media were discarded, and the cells were washed with PBS three times. The cells were collected and lysed on ice for 30 min with 500 μL of RIPA buffer (Beyotime, China) containing PMSF solution (Beyotime, China). The samples were subsequently centrifuged at 12 000 × *g* at 4 °C for 10 min. The supernatants were transferred to fresh tubes and incubated with 35 μL of α-Myc or α-GFP affinity gel (Abmart, USA) according to the manufacturer’s instructions. The protein pellets were washed three times with lysis buffer, resuspended in 50 μL of 2 × SDS sample loading buffer, boiled at 100 ℃ for 10 min, and analysed by western blotting.

### Luciferase promoter activity assay

EPC cells were seeded in 24-well cell culture plates overnight and transfected with pRL-TK (25 ng)/empty plasmid (250 ng), Flag-hnRNPC (250 ng)/ifnφ1pro (250 ng) or ISRE-Luc, followed by infection with SVCV (MOI = 1) or transfection with poly(I:C) (5 µg). After 24 h, the cells were lysed, and luciferase activity was measured with the Dual-Luciferase Reporter Assay System (Promega) according to the manufacturer’s instructions. For adaptor molecule-induced IFN/ISRE promoter activity, empty plasmid or Flag-hnRNPC (250 ng)/adaptor molecule plasmids (250 ng)/ifnφ1pro or ISRE-Luc (250 ng)/pRL-TK (25 ng) were used.

### Statistical analysis

The data were analysed using GraphPad Prism 5.0 (GraphPad Software). The significance of the dataset was determined by Student’s *t* test, and *p* < 0.05, *p* < 0.01 and *p* < 0.001 were considered statistically significant.

## Results

### HnRNPC is evolutionarily conserved and can be induced by SVCV and IFN

To determine the conservation of the hnRNPC gene in the genomes of different vertebrate phyla, including zebrafish, mice and humans, we performed synteny analysis of the hnRNPC genes. We found that the genomic organization and gene synteny of *hnRNPC* are well conserved during evolution (Additional file 1). In zebrafish, mice and humans, the *hnRNPC* genes are linked with *PARP2*, *TEP1*, *CHD8*, *TOX4* and *MMP14*. In the phylogenetic tree constructed using the hnRNPC protein sequences (Additional file 2), zebrafish hnRNPC resides in the branch containing mouse and human hnRNPC, which groups with hnRNPs. High conservation of hnRNPC proteins is also supported by sequence alignment. In addition, the genomic structures of the zebrafish and human *hnRNPC* genes are quite similar, with the last 5 exons being comparable in size.

**Figure 1:**
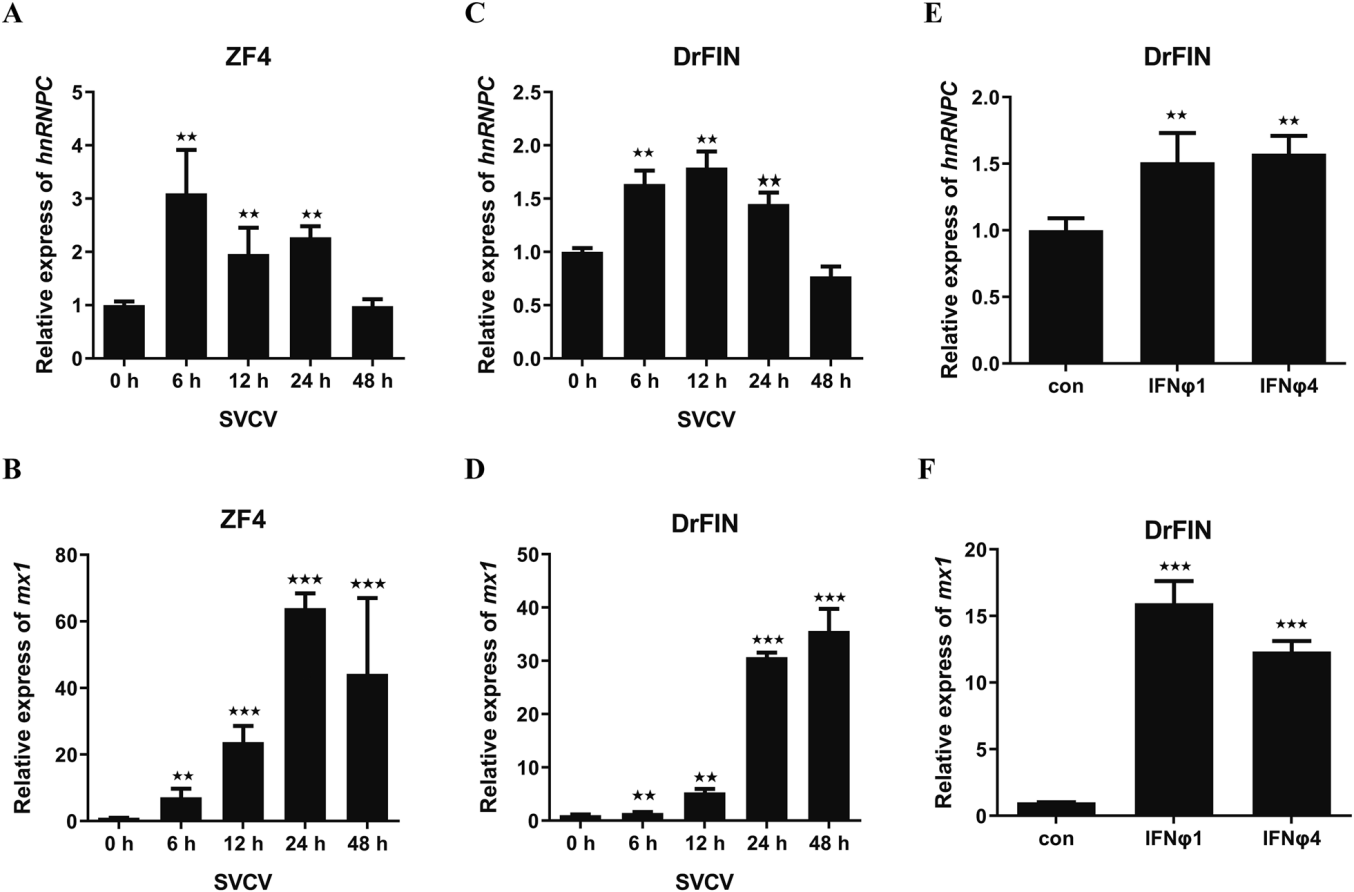
**HnRNPC is induced by SVCV or IFN**. **A**–**D** ZF4 and DrFIN cells were infected with SVCV, and the cells were collected at 6 h, 12 h, 24 h and 48 h. qPCR was used to analyse the expression of *hnRNPC* and *mx*. β-actin was used as a control. **E** and **F** qPCR analysis of *hnRNPC* and *mx* expression in DrFIN cells stimulated with IFNφ1 or IFNφ4. β-actin was used as a control. The results are shown as the mean ± SD. Asterisks indicate statistically significant differences (**p < 0.01; ***p < 0.001; *N* = 3).

To determine whether hnRNPC responds to viral infection, we examined *hnRNPC* expression in zebrafish cell lines after infection with SVCV. As shown in Figure 1A and C, the expression of *hnRNPC* significantly increased at 6, 12 and 24 h after SVCV infection and returned to basal level at 48 h. In addition, we stimulated DrFIN cells with recombinant IFNφ1 and IFNφ4 proteins, and found that both could significantly induce *hnRNPC* expression (Figure 1E). As expected, the expression of *mx* was upregulated by SVCV, poly(I:C) and IFN (Figure 1B, D and F). These results strongly suggest that hnRNPC is involved in the antiviral response.

### HnRNPC promotes virus replication

**Figure 2:**
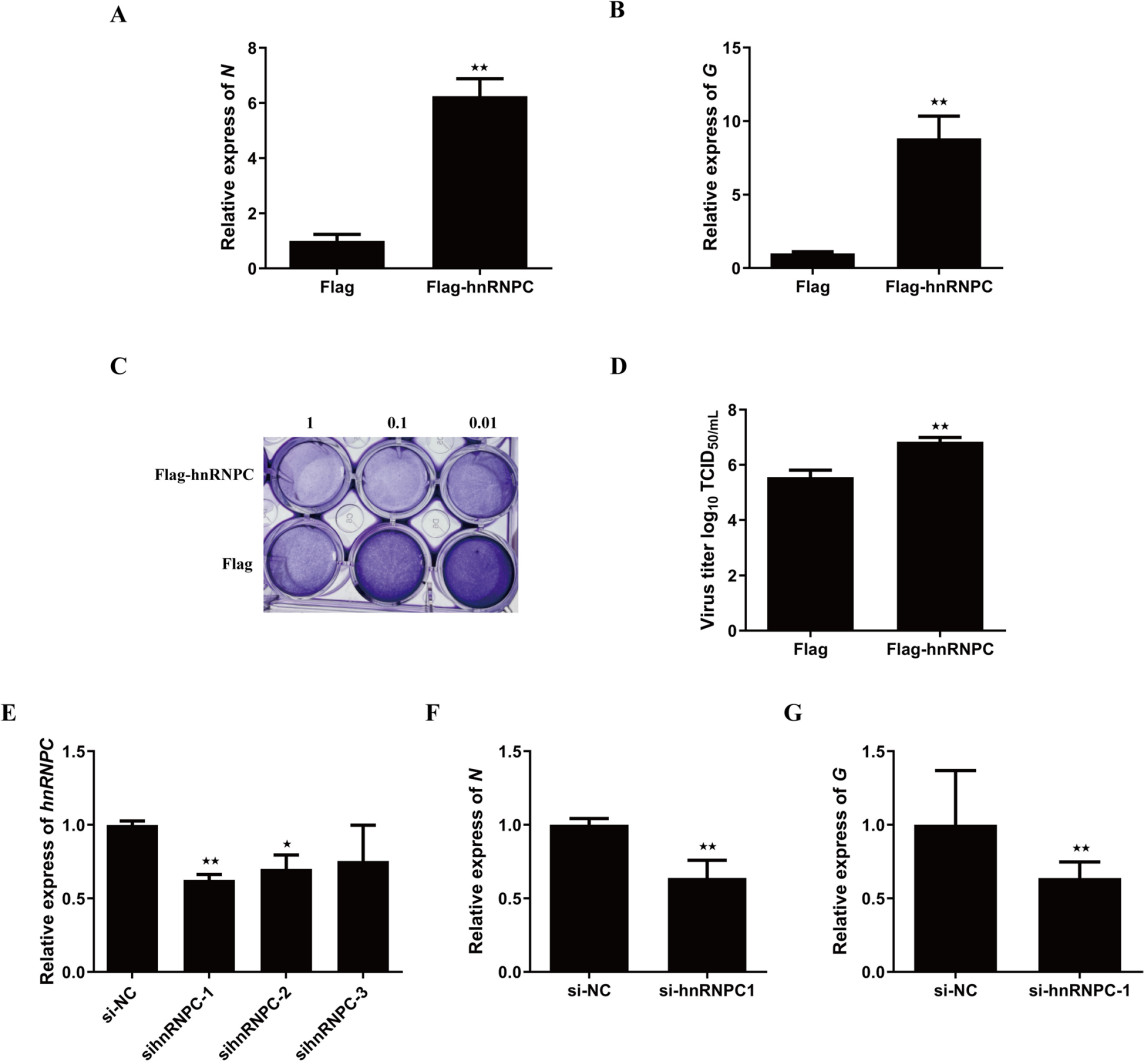
**HnRNPC negatively regulates the cellular antiviral response**. **A** and **B** EPC cells were transfected with hnRNPC and then infected with SVCV. After 24 h, the expression levels of SVCV-*N* (**A**) and SVCV-*G* (**B**) were analysed. **C** EPC cells were transfected with vector or Flag-hnRNPC and then infected with SVCV. After 24 h, the cells were fixed with 4% PFA and stained with 1% crystal violet. **D** Culture media from the cells infected with SVCV were collected, and viral titres were measured according to the TCID_50_. **E** DrFIN cells were transfected with si-NC (negative control), si-hnRNPC-1, si-hnRNPC-2 or si-hnRNPC-3. After 24 h, the expression levels of *hnRNPC* were analysed. **F** and **G** DrFIN cells were transfected with si-NC or si-hnRNPC-1 and then infected with SVCV. After 24 h, the expression levels of SVCV-*N* (**F**) and SVCV-*G* (**G**) were analysed. β-actin was used as the internal control. The results are shown as the mean ± SD. Asterisks indicate statistically significant differences (*p < 0.05; **p < 0.01; *N* = 3).

The overexpression of *hnRNPC* significantly increased the expression of the *N* (Figure 2A) and *G* (Figure 2B) genes of SVCV. The cell cytopathic effects (CPEs) (Figure 2C) and virus titres in the culture media were also significantly greater (Figure 2D) than control. Next, we tested three hnRNPC-specific small interfering RNAs (siRNAs; si-hnRNPC-1, si-hnRNPC-2, and si-hnRNPC-3) for knockdown of *hnRPC* expression and found that sihnRNPC-1 could effectively reduce the transcription level of *hnRNPC* (Figure 2E). The DrFIN cells were transfected with si-hnRNPC-1 or si-NC (negative control) and infected with SVCV for 24 h. We found that si-hnRNPC-1 significantly reduced the expression of *N* (Figure 2F) and *G* (Figure 2G) protein of SVCV. These results indicate that hnRNPC promoted SVCV replication in DrFIN cells.

### HnRNPC increases SVCV phosphoprotein (SVCV-P) stability by suppressing K48-linked polyubiquitination

**Figure 3:**
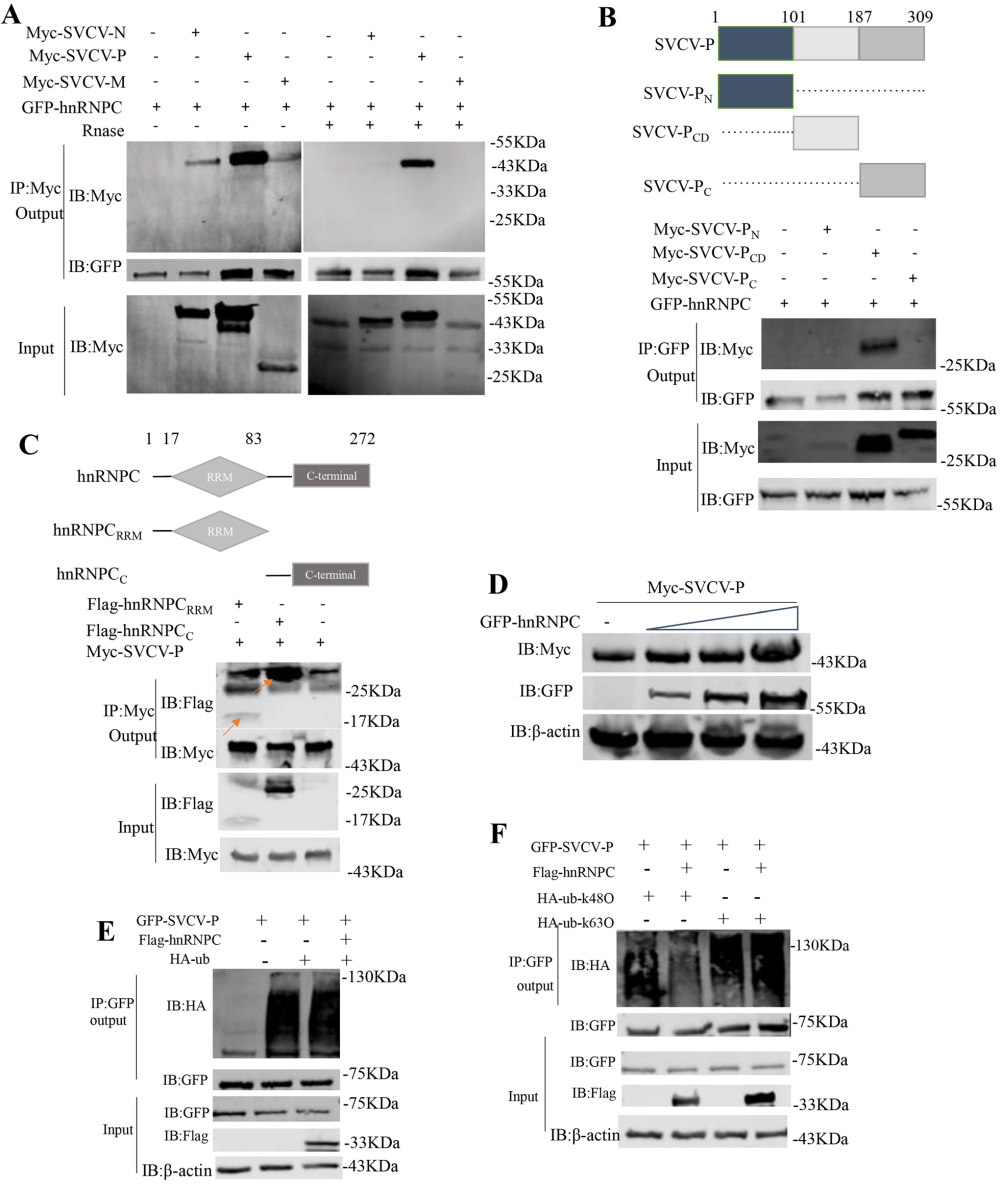
**HnRNPC increases SVCV-P stability by suppressing K48-linked polyubiquitination.** **A**–**C** HEK293 cells were transfected with the indicated plasmids. At 24 h post-transfection, the cells were harvested for Co-IP. **D** EPC cells were co-transfected with combinations of the indicated plasmids, including Myc-SVCV-P with GFP-hnRNPC (0.5, 1 or 2 µg). At 24 h post-transfection, the cells were harvested for immunoblotting. E, F HEK293 cells were co-transfected with GFP-SVCV-P, GFP-SVCV-P/HA-ub, or GFP-SVCV-P/HA-ub/Flag-hnRNPC (**E**) and HEK293 cells were co-transfected with GFP-SVCV-P/HA-ub-K48O, GFP-SVCV-P/HA-ub-K48O/Flag-hnRNPC, GFP-SVCV-P/HA-ub-K63O, or GFP-SVCV-P/HA-ub-K63O/Flag-hnRNPC (**F**). The cells were collected at 24 h post-transfection and used for the co-IP assay.

To understand the mechanisms underlying hnRNPC-mediated SVCV replication, we sought to analyse whether hnRNPC is involved in posttranslational modifications of virus structural proteins (N, P, and M). Using a coimmunoprecipitation (Co-IP) approach, we observed that GFP-hnRNPC immunoprecipitated with SVCV-N, SVCV-P, and SVCV-M in transfected HEK293 cells (Figure 3A). SVCV-P showed the highest protein binding affinity; thus, we selected SVCV-P for further investigation. Previous studies have shown that the hnRNPC protein is among the most abundant proteins in cells, and binds to RNA molecules [30]. We found that digestion of cell lysates with RNase did not affect the binding affinity of hnRNPC with SVCV-P (Figure 3A), suggesting that the interaction between hnRNPC and SVCV-P is RNA independent.

Next, we determined the protein structural domains required for the interaction between hnRNPC and SVCV-P. Several plasmids, including Myc-SVCV-P_N_, Myc-SVCV-P_CD_, Myc-SVCV-P_C_, Flag-hnRNPC_RRM_ (1–83 aa) and Flag-hnRNPC_C_ (84–272 aa), were constructed for the Co-IP assay (Figures 3B and C). We found that Myc-SVCV-P_CD_, but not Myc-SVCV-P_N_ or Myc-SVCV-P_C_, coimmunoprecipitated with GFP-hnRNPC (Figure 3B). Further analysis revealed that Myc-SVCV-P bound to both Flag-hnRNPC_RRM_ and Flag-hnRNPC_C_ (Figure 3C), indicating that 101–187 aa of SVCV-P bound to the RRM and C-terminal domain of hnRNPC. Additionally, we observed that the protein levels of SVCV-P steadily increased in a GFP-hnRNPC dose-dependent manner (Figure 3D), suggesting that hnRNPC may promote the accumulation of SVCV-P. It has been well documented that ubiquitination regulates protein degradation [15]; thus, we reason that hnRNPC may target the ubiquitin‒proteasome pathway to inhibit the degradation of SVCV-P. To test this hypothesis, HEK293 cells were cotransfected with Flag-hnRNPC, GFP-SVCV-P, and HA-tagged ubiquitin (Figure 3E). We found that hnRNPC significantly inhibited the K48-linked ubiquitination of SVCV-P; intriguingly, K63-linked ubiquitination was not reduced (Figure 3F), suggesting that hnRNPC stabilized SVCV-P by inhibiting K48-linked ubiquitination.

### HnRNPC inhibits the IFN promoter activity induced by SVCV and poly(I:C)

**Figure 4:**
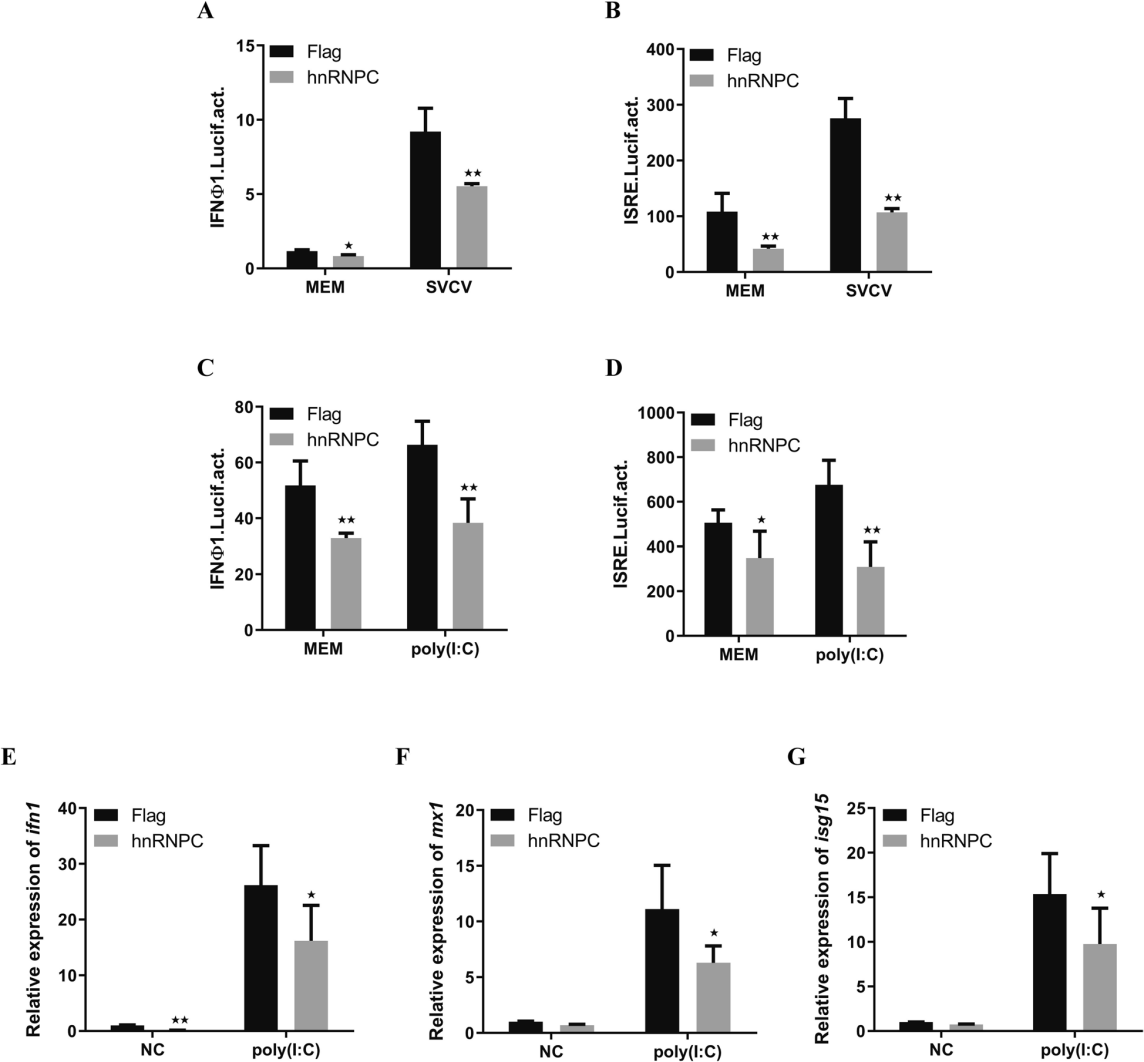
**HnRNPC inhibits ifnα1 promoter activity induced by SVCV and poly(I:C).** **A**–**D** EPC cells were co-transfected with pRL-TK (25 ng)/empty plasmid (250 ng) or Flag-hnRNPC (250 ng)/ifnφ1pro (250 ng) or ISRE-Luc (250 ng). At 24 h post-transfection, the cells were left untreated (MEM) or treated with SVCV or poly(I:C) (5 mg/mL). After 24 h, the cells were collected for the detection of luciferase activity. **E**–**G** EPC cells were transfected with vector or hnRNPC and then transfected with poly(I:C). After 24 h, qPCR was used to analyse the expression of *ifn1*, *mx* and *isg15*. β-actin was used as a control. The results are shown as the mean ± SD. Asterisks indicate statistically significant differences (*p < 0.05; **p < 0.01; *N* = 3).

Given that hnRNPC is upregulated by IFN and promotes SVCV replication, we sought to investigate whether hnRNPC is involved in modulating IFN production. A gene promoter-driven reporter assay demonstrated that the promoters of ifnφ1 and ISRE were activated in response to SVCV infection (Figures 4A and B). However, activation was significantly impaired by hnRNPC. In the case of poly(I:C) transfection, hnRNPC also displayed a similar disruptive capacity to suppress promoter activity (Figures 4C and D). Furthermore, we found that the transcription levels of genes involved in the IFN response, including *ifn1*, *mx*, and *isg15*, were reduced (Figures 4E–G). These results suggest that hnRNPC negatively regulates the production of type I IFNs.

### HnRNPC inhibits the IFN promoter activity induced by the RLR signalling pathway

**Figure 5:**
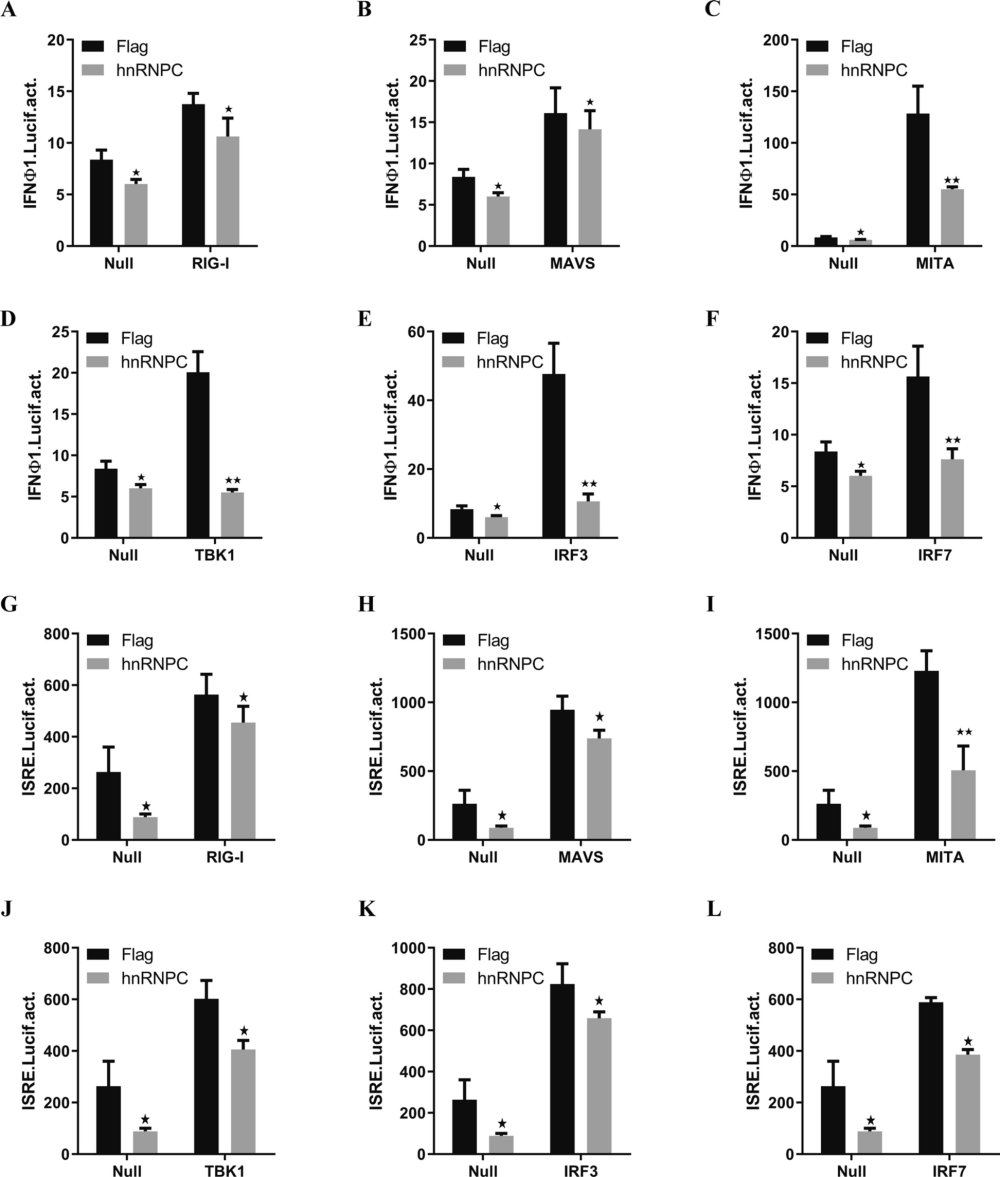
**HnRNPC inhibits ifnα1 promoter activity induced by the RLR signalling pathway.** **A**–**F** EPC cells were co-transfected with 250 ng of empty plasmid or Flag-hnRNPC/250 ng of ifnφ1pro-Luc/25 ng of pRL-TK/250 ng of empty plasmid, Flag-RIG-I, Flag-MAVS, Flag-MITA, Flag-TBK1, Flag-IRF3, or Flag-IRF7. **G**–**L** EPC cells were co-transfected with 250 ng of empty plasmid or Flag-hnRNPC/250 ng of ISRE-Luc/25 ng of pRL-TK/250 ng of empty plasmid, Flag-RIG-I, Flag-MAVS, Flag-MITA, Flag-TBK1, Flag-IRF3, or Flag-IRF7. At 24 h post-transfection, the cells were collected for the detection of luciferase activity. The results are shown as the mean ± SD. Asterisks indicate statistically significant differences (*p < 0.05; **p < 0.01; *N* = 3).

To further determine the role of hnRNPC in RLR-mediated induction of IFN expression, we overexpressed hnRNPC and RIG-I, MAVS, MITA, TBK1, IRF3, or IRF7 and examined their effects on the activities of the ifnφ1 and ISRE promoters. The overexpression of RLR factors markedly enhanced ifnφ1 and ISRE promoter activities; such activation could be inhibited by hnRNPC (Figures 5A–L). These results highlight the negative regulatory effects of hnRNPC on the transcription of *ifn* and *isg* gene activation, which are mediated by the RLR pathway.

### Interaction of hnRNPC with RLR factors triggers MITA degradation

**Figure 6:**
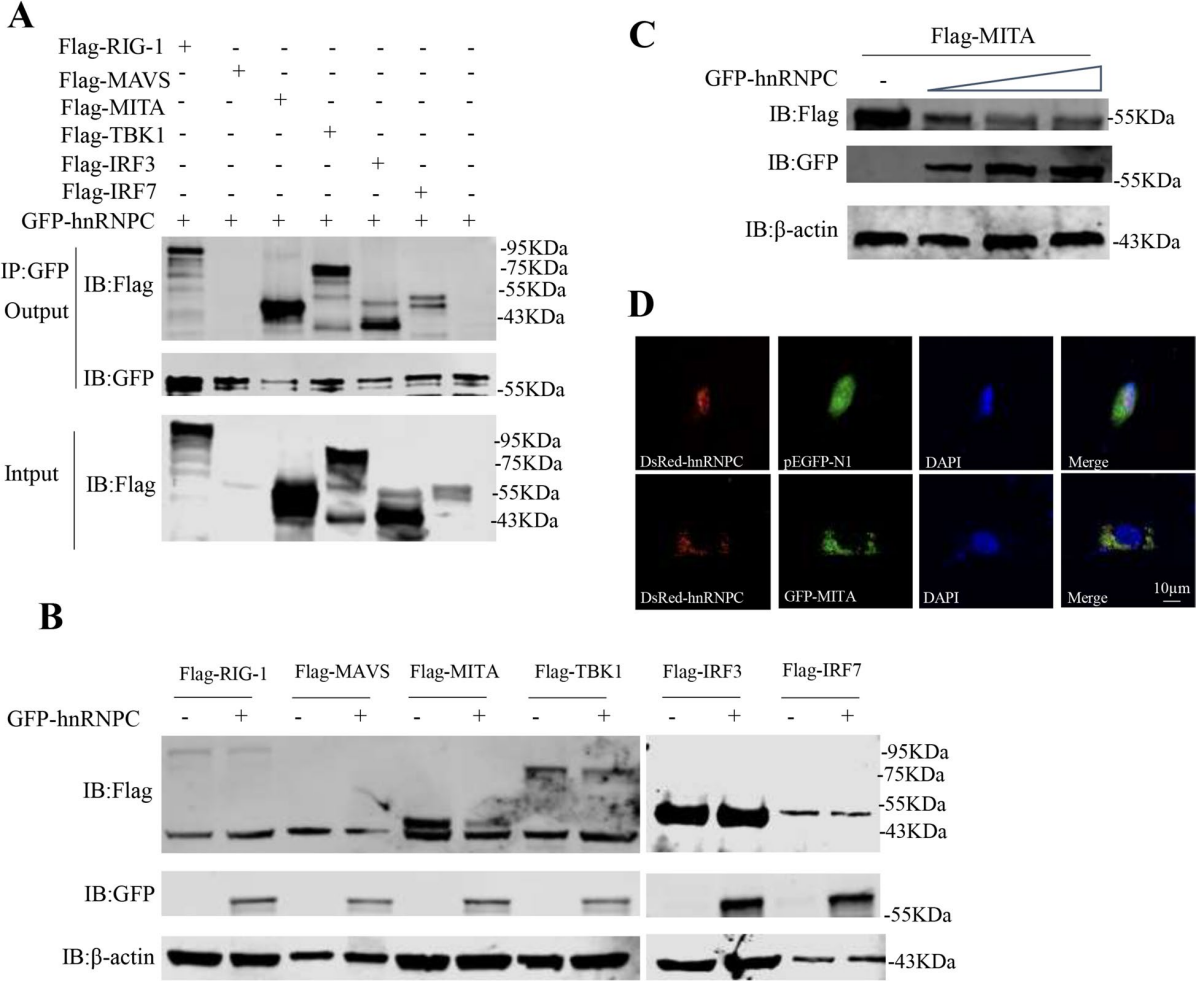
**The interaction between hnRNPC and RLR factors triggers the degradation of MITA.** **A** HEK293 cells were co-transfected with GFP-hnRNPC plus Flag-RIG-I, Flag-MAVS, Flag-MITA, Flag-TBK1, Flag-IRF3, or Flag-IRF7. The cells were collected 24 h post-transfection and used for the co-IP assay. **B** EPC cells were co-transfected with Flag-RIG-I, Flag-MAVS, Flag-MITA, Flag-TBK1, Flag-IRF3, or Flag-IRF7 plus pEGFP-N1 or GFP-hnRNPC. At 24 h post-transfection, the cells were harvested for immunoblotting. **C** EPC cells were co-transfected with Flag-MITA plus pEGFP-N1 or GFP-hnRNPC. After 24 h, the cells were harvested for immunoblotting. **D** DrFIN cells were co-transfected with DsRed-hnRNPC/pEGFP-N1 or DsRed-hnRNPC/GFP-MITA. After 24 h, the cells were subjected to fluorescence microscopy.

Posttranslational modifications affect protein stability and degradation, which involve physical contact among proteins. To investigate whether hnRNPC participates in the posttranslational modifications of RLR transcription factors, we analysed their interactions. Co-IP revealed that hnRNPC bound to RIG-I, MITA, TBK1, IRF3, and IRF7 but not to MAVS (Figure 6A). Moreover, hnRNPC overexpression decreased the protein level of MITA in EPC cells (Figures 6B and C). Confocal microscopy revealed that the degradation of MITA by hnRNPC occurred in the cytoplasm (Figure 6D). The results indicated that hnRNPC interacted with RLR factors, and induced the degradation of MITA.

hnRNPCs are highly similar in vertebrates, sharing 64% identity between human and zebrafish homologues; thus, we speculate that the functions of hnRNPCs were conserved during evolution. To characterize the effects of hnRNPC on the IFN response in humans, hnRNPC-human cells were overexpressed and transfected with poly(I:C). As shown in Additional files 3A–C, overexpression of hnRNPC-human inhibited the transcription levels of *isg15*, *pkr*, and *mx* induced by poly(I:C). Moreover, hnRNPC-human abrogated the promoter activity of IFNβ (Additional file 3D) and ISRE (Additional file 3E) and MITA-induced ISRE promoter activity (Additional file 3F). Co-IP assays revealed that hnRNPC-human could form a protein complex with MITA (Additional file 2G). We also observed that hnRNPC-human overexpression decreased the protein levels of MITA-human in HEK293 cells (Additional file 3H). Taken together, these results suggest that the inhibitory role of hnRNPC in the IFN response is conserved.

### HnRNPC mediates MITA degradation via the proteasome pathway

**Figure 7:**
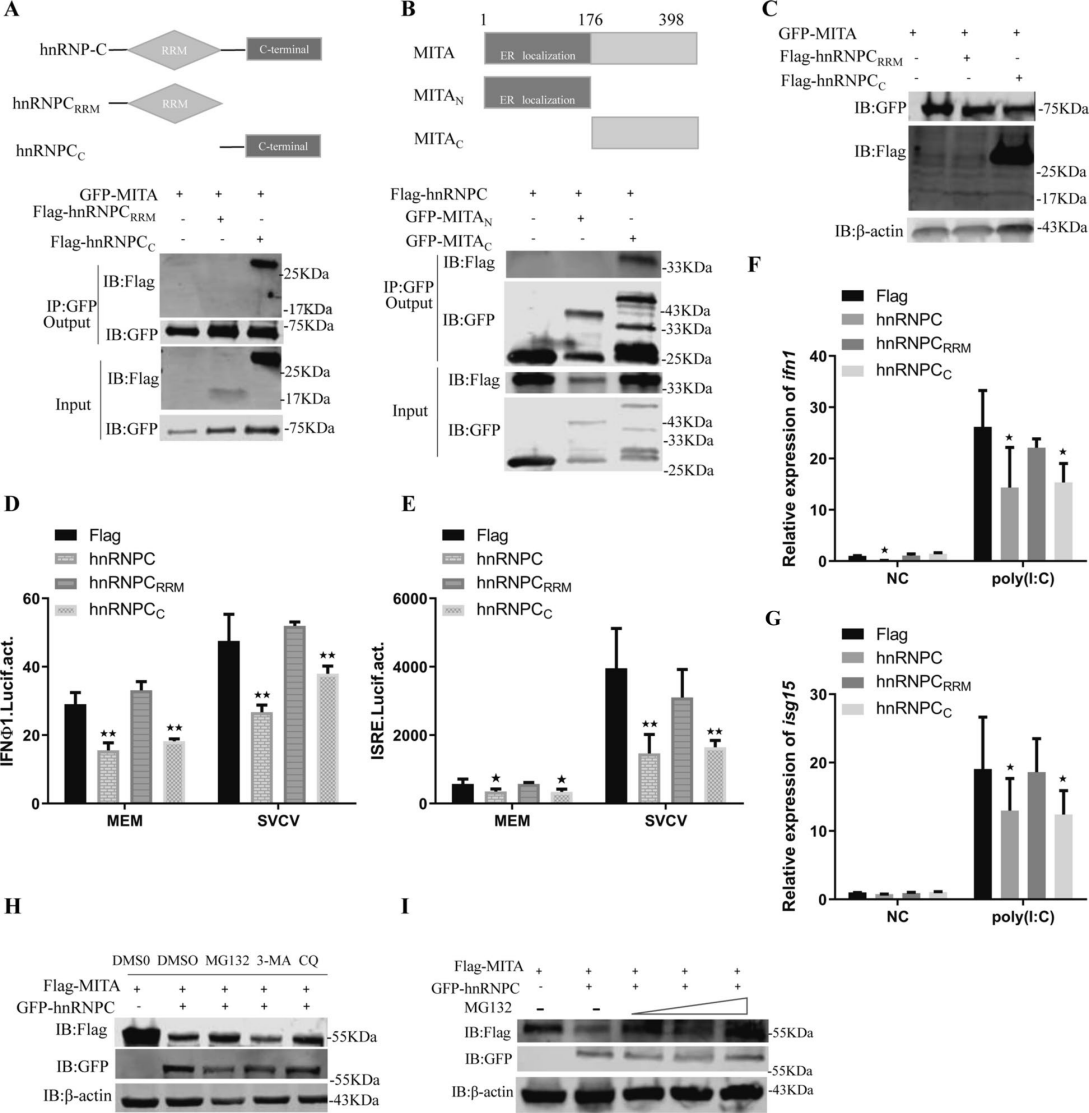
**Degradation of MITA induced by hnRNPC is mediated via the proteasome pathway.** **A**–**C** HEK293 cells were co-transfected with the indicated plasmids. At 24 h post-transfection, the cells were analysed by immunoblotting. **D**, **E** EPC cells were co-transfected with 250 ng of empty plasmid, Flag-hnRNPC, Flag-hnRNPC_RRM_, or Flag-hnRNPC_C_/250 ng of ifnφ1pro-Luc/25 ng of pRL-TK (**D**) or 250 ng of empty plasmid, Flag-hnRNPC, Flag-hnRNPC_RRM_, or Flag-hnRNPC_C_/250 ng of ISRE-Luc/25 ng of pRL-TK (**E**). At 24 h post-transfection, the cells were harvested for the detection of luciferase activity. **F**, **G** EPC cells were co-transfected with vector, Flag-hnRNPC, Flag-hnRNPC_RRM_, or Flag-hnRNPC_C_ and then transfected with poly(I:C). qPCR was used to analyse the expression of *ifn1* and *isg15*. **H**, **I** EPC cells were co-transfected with Flag-MITA and either pEGFP-N1 or GFP-hnRNPC. After 6 h, the cells were treated with DMSO, MG132 (25 μM), 3-MA (10 mM), CQ (50 μM) (**I**) or 3-MA (5 mM, 10 mM or 15 mM). After 24 h, the cells were collected for immunoblotting. The results are shown as the mean ± SD. Asterisks indicate statistically significant differences (*p < 0.05; **p < 0.01; *N* = 3).

To determine the structural domain of hnRNPC required for the interaction with MITA, HEK293 cells were cotransfected with GFP-MITA plus Flag-hnRNPC_RRM_ or Flag-hnRNPC_C_. MITA coimmunoprecipitated with Flag-hnRNPC_C_ but not with Flag-hnRNPC_RRM_ (Figure 7A). Two construct mutants containing the N-(GFP-MITA_N_) or C-(GFP-MITA_C_) terminal region were constructed (Figure 7B). We found that GFP-MITA_C_, but not GFP-MITA_N_, coimmunoprecipitated with Flag-hnRNPC (Figure 7B), indicating that the C-terminal domain of MITA bound to the C-terminal domain of hnRNPC. Moreover, the overexpression of Flag-hnRNPC_C_, but not of Flag-hnRNPC_RRM_, decreased the MITA protein in the transfected cells relative to that in the control cells (Figure 7C). Notably, Flag-hnRNPC_C_ significantly inhibited the promoter activities of ifnφ1pro (Figure 7D) and ISRE (Figure 7E) induced by SVCV. Furthermore, the transcription levels of IFNs, including *ifn1* and *isg15*, were reduced (Figures 7F and G). To determine the signalling pathway mediating hnRNPC-induced MITA degradation, we treated the transfected cells with MG132 (a proteasome inhibitor), 3-MA (an autophagy inhibitor), or CQ (a lysosomal inhibitor). We found that hnRNPC-mediated MITA degradation could be mostly rescued in a dose-dependent manner by MG132 but not 3-MA or CQ (Figures 7H and I), suggesting that the proteasome pathway is mainly responsible for the hnRNPC-induced degradation of MITA.

### HnRNPC enhances the K48-linked polyubiquitination of MITA

**Figure 8:**
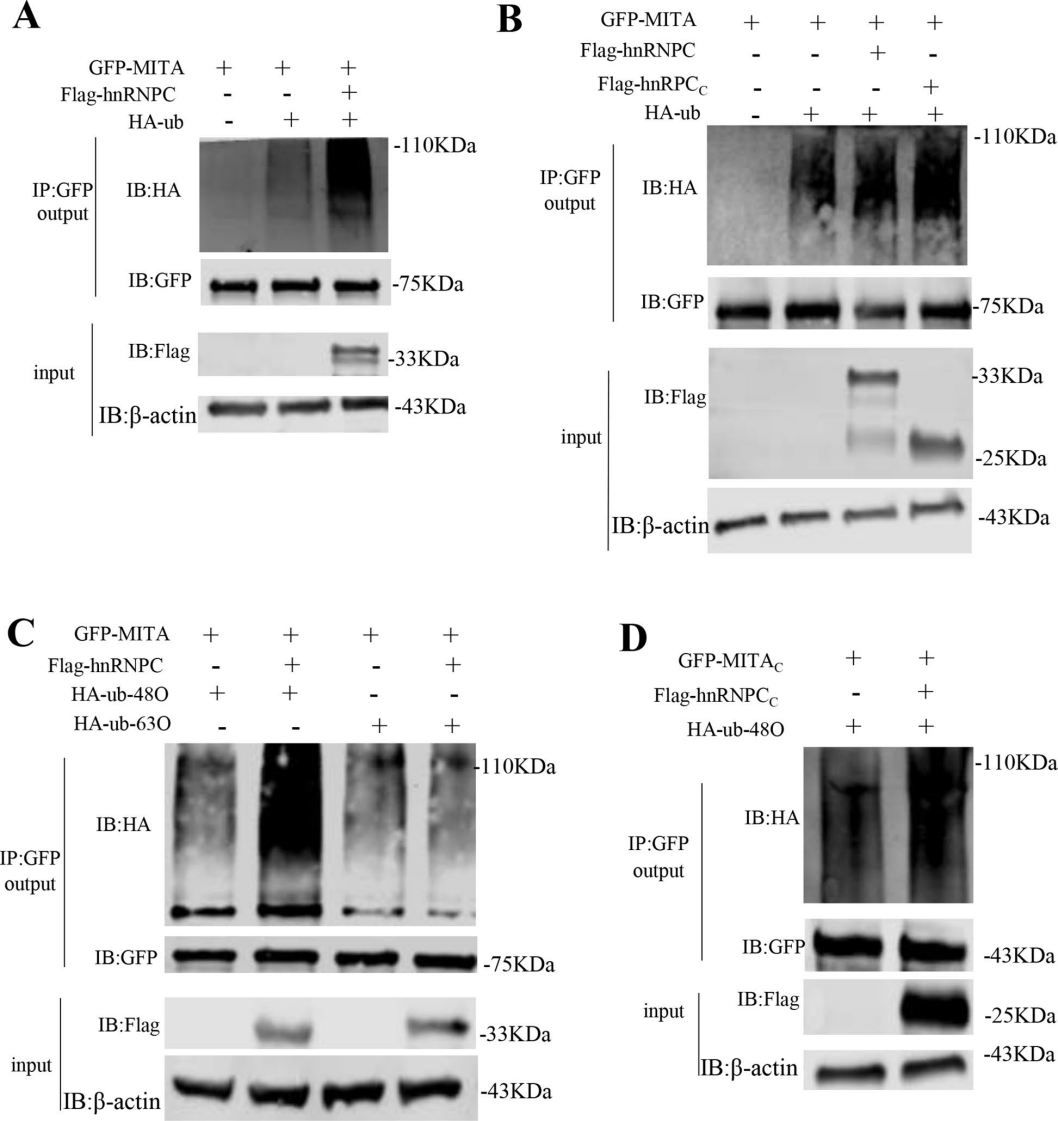
**HnRNPC enhanced K48-linked polyubiquitination of MITA.** **A**–**D** HEK293 cells were co-transfected with the indicated plasmids. The cells were collected 24 h post-transfection and used for the co-IP assay.

To understand the mechanism of hnRNPC in the degradation of MITA, we sought to determine whether hnRNPC induces polyubiquitination of MITA and what type of ubiquitin chain is catalyzed by hnRNPC. We found that hnRNPC participated in the ubiquitination of GFP-MITA (Figure 8A), which was dependent on the hnRNPC domain (Figure 8B). We also observed that hnRNPC overexpression significantly promoted the K48-linked ubiquitination of MITA but not the K63-linked ubiquitination (Figure 8C). Moreover, hnRNPC elevated the K48-linked ubiquitination of the MITA C-terminal domain (MITA_C_) (Figure 8D). These data suggest that hnRNPC catalyzes the K48-linked ubiquitination of the MITA_C_ domain, resulting in MITA proteasomal degradation.

## Discussion

HnRNPs are a family of diverse RNA-binding proteins involved in RNA metabolism, consisting of 20 members with molecular weights ranging from 34 to 120 kDa [19]. Emerging evidence suggests that members of the hnRNP family participate in virus replication; however, whether they favour or suppress virus propagation remains controversial. For example, hnRNPA1 and A2 bind to virus RNA and are shown to be required for the translation of viral components [26, 40–42]. In addition, hnRNPK promotes the replication of vesicular stomatitis virus by inhibiting the apoptosis of infected cells and maintaining virion stability [43]. Conversely, hnRNPE1 and E2 interact with vesicular stomatitis virus phosphoprotein and inhibit virus replication by reducing viral gene expression [44]. Recent studies have shown that hnRNPC binds to a variety of viral proteins, such as poliovirus [45], influenza A [32], dengue virus [33, 46], and hepatitis delta virus [47], to either promote or suppress virus replication. However, the mechanism underlying the functions of hnRNPC in antiviral innate immunity is still unclear. In this study, we demonstrated that hnRNPC interacts with SVCV-P to increase its stability, thereby increasing the availability of virions for virus replication (Figure 3).

Several studies have revealed that hnRNPC plays an important role in viral infection. HnRNPC interacts with nuclear proteins via its C-terminal auxiliary domain and negatively regulates influenza virus replication [32]. HnRNPC1/C2 interacts with vimentin and NS1 of Dengue virus (DENV) and are involved in DENV replication [46]. In addition, hnRNPC1/C2 binds to the RNA of poliovirus and is essential for the synthesis of viral positive-strand RNA [48]; however, the specific mechanism by which hnRNPC regulates the virus remains unknown. In this study, we demonstrated that hnRNPC interacts with SVCV-P to promote viral proliferation by inhibiting K48-linked ubiquitination to maintain the stability of the P protein.

Type I IFNs play a central role in the innate immune defense against virus infection. The production of IFNs involves multiple signalling events that are regulated by RLR transcription factors and are tightly controlled to induce the expression of antiviral effectors and to avoid damage caused by overproduction. This regulatory network is currently known; for example, FBXO3, which belongs to the F-box family of proteins, is involved in host autoimmune and inflammatory responses and can catalyse the K27-linked ubiquitination of IRF3 and IRF7, leading to their proteasomal degradation and thereby inhibiting the IFN response [49]. Guanylate-binding protein 4, a member of the superfamily of GTPases that are abundantly expressed in animal cells as dynamin and Mx proteins, inhibits IFN production by disrupting the formation of the TRAF6-IRF7 complex [50]. In this study, we showed that hnRNPC interacted with several key molecules for IFN expression, including RIG-1, MITA, TBK1, IRF3, and IRF7 (Figure 5). Intriguingly, hnRNPC alone enhanced the degradation of MITA to downregulate IFN expression, indicating that MITA is the main target of hnRNPC (Figure 6). Moreover, we found that the negative regulatory roles of hnRNPC are conserved in zebrafish and humans. Collectively, our findings identified hnRNPC as a suppressor of IFN response, expanding the existing repertoire of IFN regulators in vertebrates.

Ubiquitination is important for protein metabolism and function. As a key mediator orchestrating IFN production, MITA stability and functions are affected by ubiquitination and deubiquitination. The tripartite motif (TRIM) family is a large class of proteins with E3 ubiquitin ligase activity. They are involved in different cellular functions and play important roles in host antiviral immune response. TRIM32 promotes the antiviral response of cells through K63-linked ubiquitination targeting MITA and by promoting the interaction between MITA and TBK1 [51]. USPs belong to the deubiquitinating enzyme superfamily and are important mediators of IFN response. USP18 recruits USP20 to catalyze K48-linked ubiquitination to promote the degradation of MITA, thereby downregulating the antiviral response [52]. Moreover, USP49 dissociates the K63-linked ubiquitin chain from MITA after herpes simplex virus type 1 infection, blocking MITA aggregation and subsequently recruiting TBK1 to the signalling complex for the activation with IRF3 and IRF7 [15]. The present study demonstrated that hnRNPC was also involved in the ubiquitination of MITA. Importantly, hnRNPC-induced ubiquitination of MITA resulted in protein degradation, which relied on the proteasome pathway to catalyze the K48-linked ubiquitination of the MITA C-terminal region (Figures 8C and D). This finding reveals a novel function of hnRNPC, previously unreported in protein posttranslational modifications and antiviral immunity.

A common strategy for viruses is to evade the innate immune system by hijacking host factors to block type I IFN production. The interaction between the neuraminidase protein NA of the influenza A virus and heat shock protein 90 (Hsp90) is essential for maintaining the stability of virions for virus replication [53]. In the case of viruses associated with foot and mouth disease, the capsid protein VP1 activates the MAPK pathway to sustain virus replication via the ribosomal protein SA [54]. In our recent studies, we reported that SVCV-P cooperates with IRF2 to increase the expression of virus proteins and negative IFN regulators in cells [38]. Furthermore, SVCV-P was shown to function as a decoy substrate for cellular TBK1, reducing IRF3 phosphorylation and IFN expression [55].

**Figure 9:**
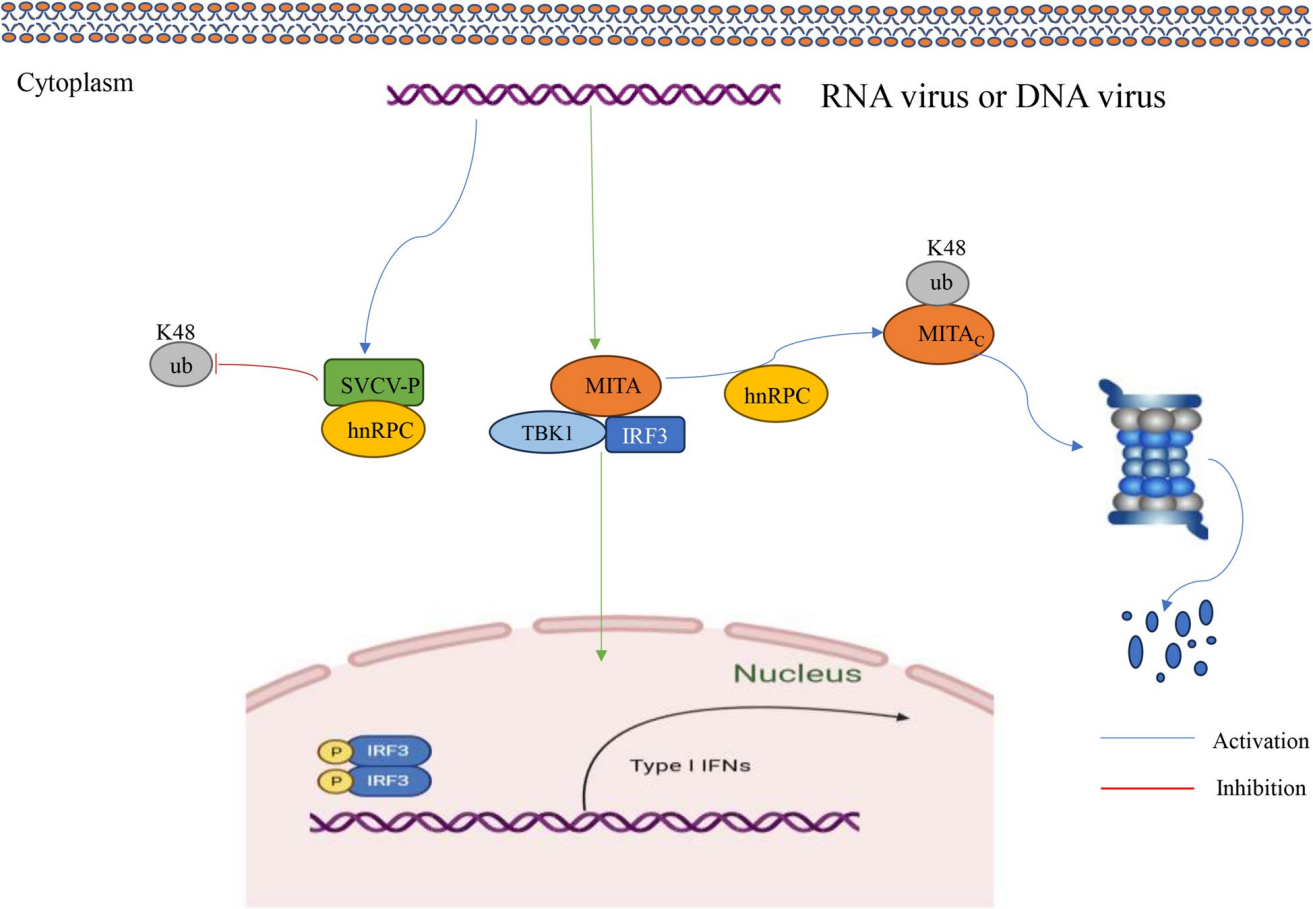
**Mechanism of HnRNPC regulation of the host antiviral response**. HnRNPC inhibited the K48-linked ubiquitination of SVCV-P to increase its stability, thereby promoting virus replication. In addition, hnRNPC is a negative regulator of type I IFN production that targets MITA, resulting in K48-linked ubiquitination and protein degradation.

In summary, we found that hnRNPC inhibited the K48-linked ubiquitination of SVCV-P to increase its stability, thereby promoting virus replication. Furthermore, we demonstrated that hnRNPC is a negative regulator of type I IFN production that targets MITA, resulting in K48-linked ubiquitination and protein degradation. Conversely, hnRNPC also increases the stability of SVCV-P to favour SVCV replication. Our data demonstrate that hnRNPC both serves as a negative regulator of host antiviral response and promotes virus replication by increasing the availability of viral proteins (Figure 9). Therefore, hnRNPC is an important player in regulating IFN response and antiviral immunity. Our work reveals a novel mechanism by which hnRNPC regulates viral replication through protein posttranslational modifications and provides valuable information for the development of antiviral therapies and vaccines.

## Supplementary Information

**Additional file 1.** **HnRNPC is evolutionarily conserved**. **A** Phylogenetic trees were generated from different species of vertebrate hnRNPs. **B** Gene synteny of hnRNPC genes. **C** Multiple sequence alignment of hnRNPC in humans, mice, and zebrafish. **D** Gene organization of hnRNPC.

**Additional file 2**. **Information on the gene sequences used for bioinformatics analysis.**

**Additional file 3.** **The inhibitory role of hnRNPC on IFN response is conserved in humans**. **A**–**C** HEK293 cells were transfected with pcDNA3.1 or Myc-hnRNPC. At 24 h post-transfection, cells were transfected with poly(I:C). After 24 h, cells were collected for the detection of luciferase activity. **D**, **E** HEK293 cells were co-transfected with 250 ng IFNβ-Luc or ISRE-Luc/250 ng pcDNA3.1, or Myc-hnRNPC-human/25 ng pRL-TK. At 24 h post-transfection, cells were left untreated (negative control) or transfected with poly(I:C). After 24 h, cells were collected for the detection of luciferase activity. **F** HEK293 cells were co-transfected with 250 ng ISRE-Luc/250 ng GFP-MITA-human, or pEGFP-N1/pcDNA3.1 or Myc-hnRNPC-human/25 ng pRL-TK. **G** HEK293 cells were co-transfected with GFP-MITA plus pcDNA3.1 or Myc-hnRNPC-human. The cells were collected 24 h post-transfection and used for Co-IP assay. **H** HEK293 cells were co-transfected with GFP-MITA-human plus pcDNA3.1 or Myc-hnRNPC-human. At 24 h post-transfection, the cells were harvested for immunoblotting. The results are shown as mean ± SD. Asterisks indicate statistically significant differences (*P < 0.05; **P < 0.01; *N* = 3).

## Data Availability

All the data generated or analysed during this study are included in this published article and its supplementary information files.
