# Supplementary figures and images for: HnRNPC triggers the degradation of MITA to suppress the interferon-mediated antiviral response
Source: Vet Res. 2025 Feb 24;56:45. doi: 10.1186/s13567-025-01463-6 (PMC11854013; doi:10.1186/s13567-025-01463-6)

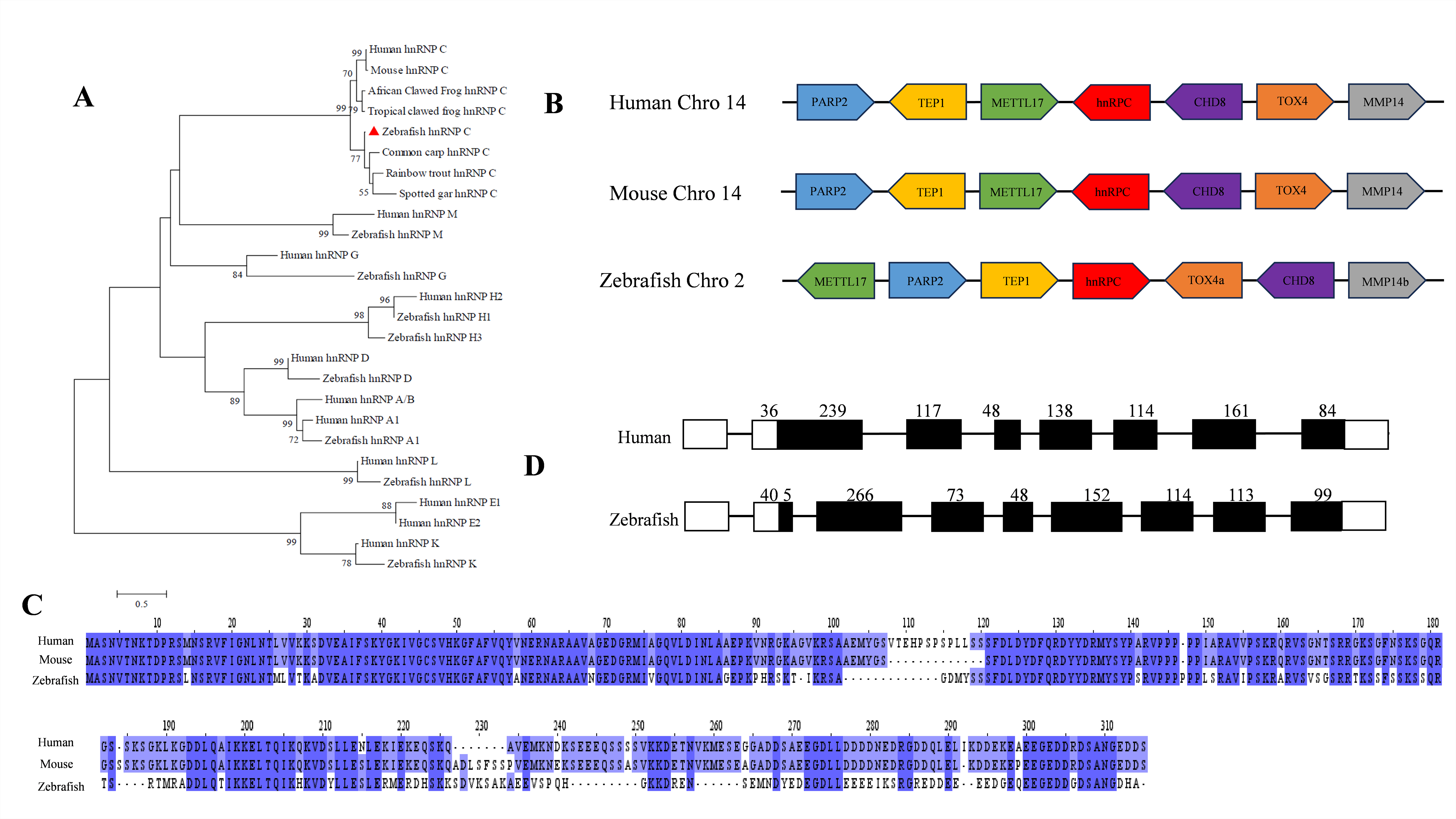

Supplement: Supplementary file 1 — Additional file 1. HnRNPC is evolutionarily conserved. A Phylogenetic trees were generated from different species of vertebrate hnRNPs. B Gene synteny of hnRNPC genes. C Multiple sequence alignment of hnRNPC in humans, mice, and zebrafish. D Gene organization of hnRNPC. [file 13567_2025_1463_MOESM1_ESM.tif]

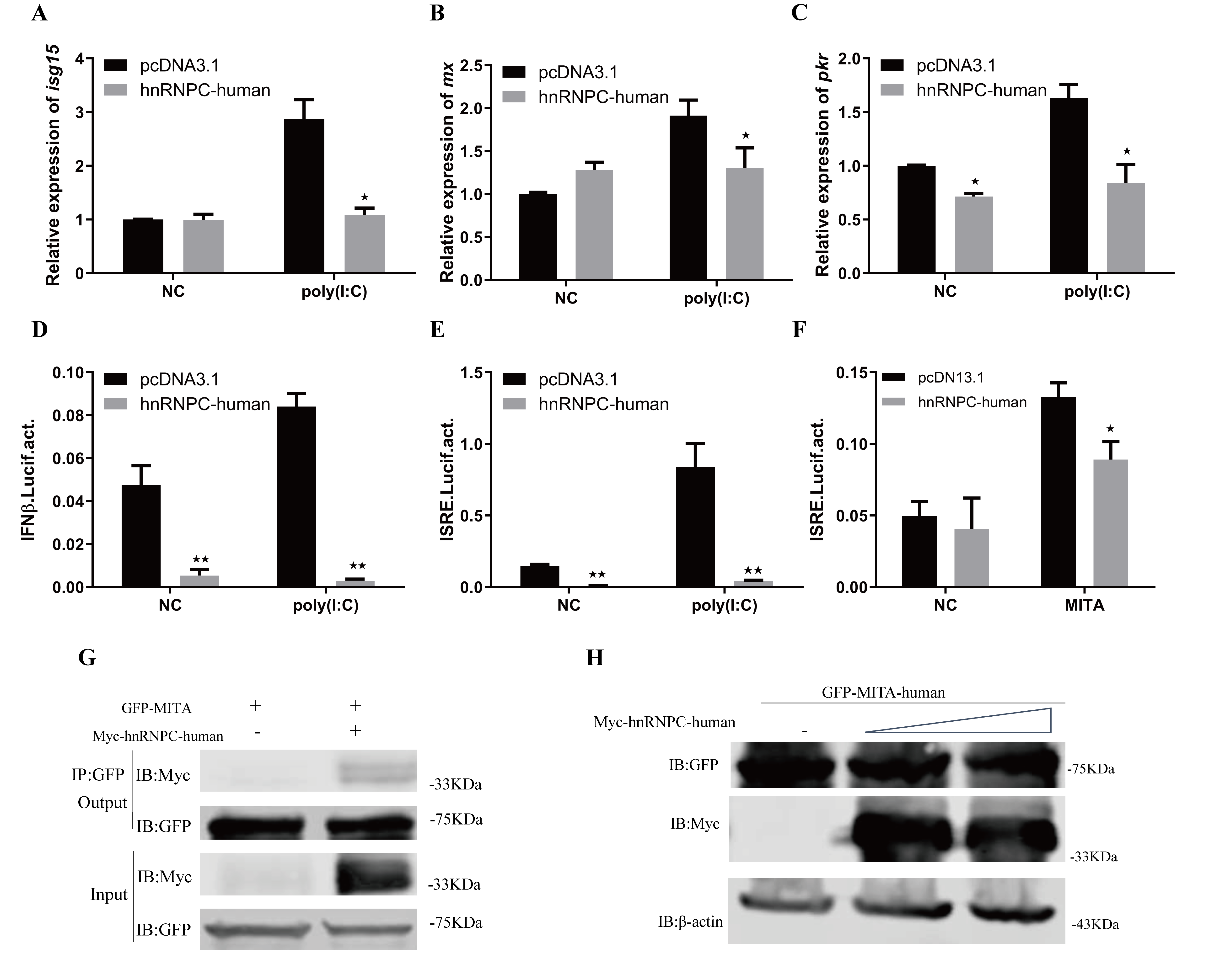

Supplement: Supplementary file 3 — Additional file 3. The inhibitory role of hnRNPC on IFN response is conserved in humans. A–C HEK293 cells were transfected with pcDNA3.1 or Myc-hnRNPC. At 24 h post-transfection, cells were transfected with poly(I:C). After 24 h, cells were collected for the detection of luciferase activity. D, E HEK293 cells were co-transfected with 250 ng IFNβ-Luc or ISRE-Luc/250 ng pcDNA3.1, or Myc-hnRNPC-human/25 ng pRL-TK. At 24 h post-transfection, cells were left untreated (negative control) or transfected with poly(I:C). After 24 h, cells were collected for the detection of luciferase activity. F HEK293 cells were co-transfected with 250 ng ISRE-Luc/250 ng GFP-MITA-human, or pEGFP-N1/pcDNA3.1 or Myc-hnRNPC-human/25 ng pRL-TK. G HEK293 cells were co-transfected with GFP-MITA plus pcDNA3.1 or Myc-hnRNPC-human. The cells were collected 24 h post-transfection and used for Co-IP assay. H HEK293 cells were co-transfected with GFP-MITA-human plus pcDNA3.1 or Myc-hnRNPC-human. At 24 h post-transfection, the cells were harvested for immunoblotting. The results are shown as mean ± SD. Asterisks indicate statistically significant differences (*P < 0.05; **P < 0.01; N = 3). [file 13567_2025_1463_MOESM3_ESM.tif]
