# Supplementary material for: HnRNPC triggers the degradation of MITA to suppress the interferon-mediated antiviral response
Source: Vet Res. 2025 Feb 24;56:45. doi: 10.1186/s13567-025-01463-6 (PMC11854013; doi:10.1186/s13567-025-01463-6)
Supplement: Supplementary file 2 — Additional file 2. Information on the gene sequences used for bioinformatics analysis. [file 13567_2025_1463_MOESM2_ESM.pdf]

Gene	Species	Accession
HnRNP A1	*Human*	NP_002127.1
	Zebrafish	NP_956398.1
HnRNP A2B1	*Human*	NP_002128.1
HnRNP A/B	*Zebrafish*	XP_005169634.1
HnRNP C	*Human*	NP_001070910.1
	Mus musculus	NP_001164452.1
	Zebrafish	NP_957287.1
	Common carp	XP_042574598.1
	Rainbow trout	XP_036845101.1
	Spotted gar	XP_006630258.1
	African clawed frog	NP_001081360.1
	Tropical clawed frog	NP_001005088.1
HnRNP D	*Human*	NP_001003810.1
	Zebrafish	XP_005155604.1
HnRNP E1	*Human*	NP_006187.2
HnRNP E2	*Human*	NP_001092090.1
HnRNP E3	*Zebrafish*	NP_001018567.1
HuRNP G	*Human*	NP_001158275.1
	Zebrafish	XP_017214473.1
HnRNP H2	*Human*	NP_001027565.1
HnRNP H1	*Zebrafish*	XP_005169638.1
HnRNP H3	*Zebrafish*	XP_005156938.1
HnRNP K	*Human*	NP_001305115.1
	Zebrafish	XP_005167376.1
HnRNP L	*Human*	NP_001005335.1
	Zebrafish	XP_005169750.1
HnRNP M	*Human*	NP_001284347.1
	Zebrafish	XP_005170511.1
